# Quantitative comparison of fluorescent proteins using protein nanocages in live cells

**DOI:** 10.1242/jcs.263858

**Published:** 2025-05-21

**Authors:** Giulia Viola, Yasmeen W. Ibrahim, Kyle A. Jacobs, Joël Lemière, Matthew L. Kutys, Torsten Wittmann

**Affiliations:** Department of Cell and Tissue Biology, University of California, San Francisco, San Francisco, CA 94143, USA

**Keywords:** Fluorescent protein comparison, Protein nanocages, mStayGold, mScarlet, Live-cell fluorescence microscopy, Intracellular diffusion

## Abstract

To standardize comparison of fluorescent protein performance on a molecule-by-molecule basis in a physiological intracellular environment, we constructed fluorescent protein-tagged I3-01 peptides that self-assemble into stable 60-subunit dodecahedrons inside live mammalian cells. We were especially interested in determining which of the recently published monomeric StayGold variants is best for live microscopy in mammalian cells. Combining nanocage brightness and photobleaching measurements into a single metric, mStayGold stood out as far superior to all other green and red fluorescent proteins we tested with a functional lifetime that is at least 8–10-fold longer compared with EGFP or mEmerald. Analysis of intracellular nanocage diffusion further confirmed the monomeric nature of mStayGold, and we demonstrate that mStayGold-tagged nanocages can serve as highly photostable nanoparticles to analyze intracellular biophysical properties. Analysis of frequently used red fluorescent proteins was less encouraging and recent mScarlet or mRuby variants did not perform substantially better than mCherry on a typical spinning disc confocal microscope system, highlighting the importance of a standardized method to benchmark fluorescent proteins to make optimal choices for specific experimental setups.

## Teaser

**Highlighted Article:** Fluorescent protein-tagged nanoparticles are used as tools to quantitatively compare fluorescent protein performance in live-cell microscopy experiments, showing that mStayGold is by far brightest and most photostable fluorescent protein available.

## INTRODUCTION

**Fig. 1.**
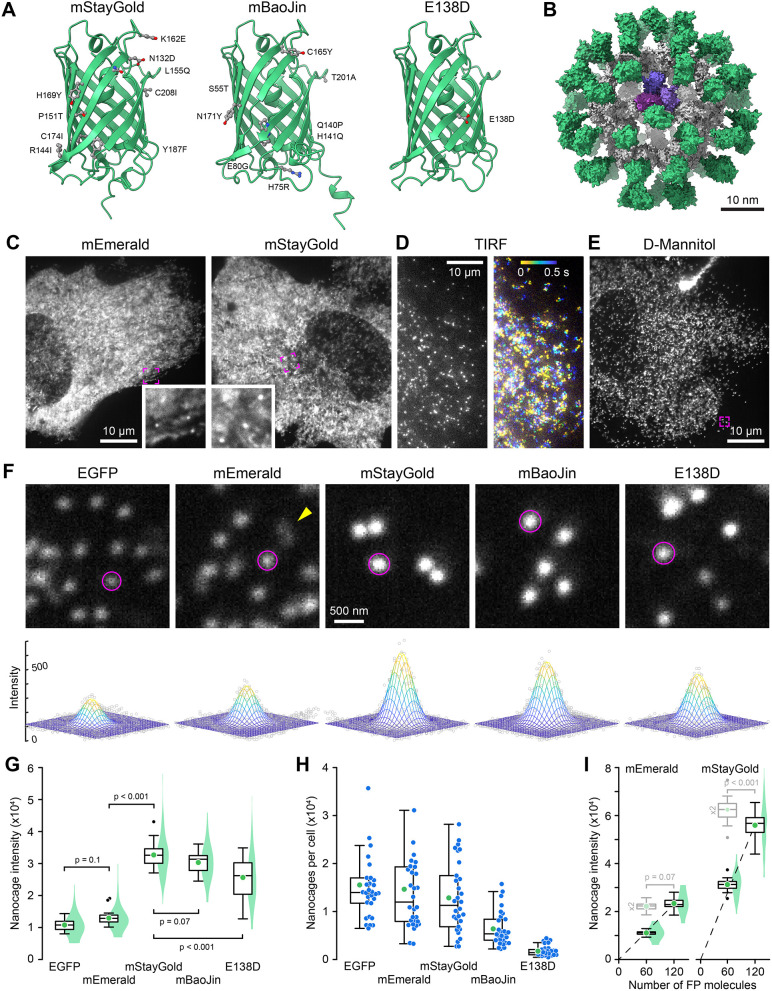
**Green FP-tagged I3-01 nanocage fluorescence intensity.** (A) AlphaFold2 structures of the three monomeric mStayGold variants viewed from the dimerization interface. Amino acid substitutions compared to the original StayGold dimer are highlighted. (B) AlphaFold2 model of an mEmerald-tagged nanocage. The I3-01 peptides are gray with one trimer highlighted in shades of purple. (C) Spinning disc confocal images of mEmerald- and mStayGold nanocage-expressing RPE cells with a 500 ms exposure time. Insets show indicated regions at higher magnification highlighting immobile nanocages. (D) mStayGold nanocages in TIRF microscopy with a 10 ms exposure time. The right panel shows a color-coded projection over 500 ms showing diffusion during this time. (E) mStayGold nanocages by spinning disc confocal microscopy in a 400 mOsm D-mannitol-treated RPE cell. The indicated region is shown at higher magnification in the middle panel below. (F) Comparable peripheral cell regions of D-mannitol-treated RPE cells expressing the indicated green FP nanocage fusions. Circles indicate areas within a two standard deviation radius as determined by 2D Gaussian fit. Bottom panels show the Gaussian fit for these example nanocage particles. Nanocages that are blurred because they moved during acquisition (yellow arrowhead) were not included in the analysis. (G) Comparison of the integrated intensity of nanocage particles tagged with the indicated FP. *n*=30 cells each from three independent experiments. Statistical analysis by one-way ANOVA with Tukey–Kramer honestly significant difference (HSD) test. (H) Estimated number of nanocages per analyzed cell calculated as total intensity per cell divided by nanocage intensity multiplied by 0.86 because the two standard deviation circle encompasses 86% of the nanocage signal of the same cell population as in G. (I) Comparison of the integrated intensity of mEmerald and mStayGold nanocages containing either 60 or 120 FP molecules. The dashed lines are linear regressions through the origin. Box plots in light gray are the 60 FP molecule nanocage intensity data multiplied by two indicating the expected distribution if the 120 FP molecule nanocages were exactly twice as bright. Statistical comparison of these expected distributions to the 120 FP molecule nanocage intensities by unpaired two-tailed Student's *t*-test. *n*=20 cells per condition. Images in C–F representative of three experimental repeats.  In C, E and F, all spinning disc confocal images were acquired with the same exposure settings and images are scaled identically with the same gray level window to allow side-by-side comparison. In G–I, green circles indicate the mean, blue symbols are data from individual cells, and violin plots (G and I) show the intensity distribution of all nanocage particles (*n*=300 per condition). Box plots show median, first and third quartile, with whiskers extending to observations within 1.5 times IQR from the quartile 1 and 3 boundaries.

Fluorescent proteins (FPs) have transformed cell biology by allowing real-time observation of dynamic biological processes, from entire organisms to individual proteins. However, photobleaching and phototoxicity pose crucial limitations, particularly during single-molecule imaging experiments or high-resolution live microscopy studying intracellular protein dynamics at low physiological expression levels, limiting the number of photons that can be collected before the signal disappears (Ettinger and Wittmann, 2014). Thus, the recent discovery of a new green FP variant from the cnidarian *Cytaeis uchidae* that, compared with *Aequorea victoria* green FPs, appears several-fold brighter and more photostable could be a game changer for live microscopy approaches (Hirano et al., 2022, 2024). However, this original StayGold is an obligate dimer limiting its usefulness for protein tagging. Motivated by this challenge, three different groups recently published very different versions of monomeric StayGold (Fig. 1A). Based on the crystal structure of the dimer, the original StayGold discoverers used targeted mutagenesis to isolate mStayGold, with nine amino acid substitutions mostly near the dimerization interface (Ando et al., 2023). Another group used random mutagenesis and screening to generate bright monomeric StayGold variants, and isolated mBaoJin, in which eight amino acid changes are more broadly distributed throughout the structure (Zhang et al., 2024). Finally, a third group identified a specific glutamate residue in the dimerization interface and generated a variant with a single E138D substitution to disrupt StayGold dimerization (Ivorra-Molla et al., 2023). This trifecta of monomeric StayGold variants was published within months of one another and raises the question of which is the objectively best variant for a particular experimental approach.

Although *in vitro* spectra of purified FPs at a known concentration document intrinsic photophysical properties, *in vitro* data likely do not fully reflect FP performance in live mammalian cells, as purified proteins in solution do not recapitulate the physiological intracellular environment. In addition, fluorescence microscopes only capture a limited band of the emission spectrum for an FP and often do not excite at the optimal wavelength, which both affects apparent brightness and photobleaching kinetics. Methods to directly evaluate FPs in live cells are limited. The total intensity of FP-expressing cells is meaningless as it is largely a measure of expression level and not a valid comparison of FP brightness. To normalize for expression, FPs of interest are often expressed together with a different wavelength reference FP from a single transcript connected by self-cleaving 2A peptide tags aiming for equimolar concentrations (Bindels et al., 2017; Ando et al., 2023; Gadella et al., 2023). The fluorescence ratio between the two FPs can account for differences in expression levels allowing more accurate comparison of relative brightness in an intracellular context. However, caveats include incomplete cleavage of the 2A peptide, which can affect fluorescence through non-radiative processes, such as fluorescence resonance energy transfer (FRET), as well as differences in maturation or degradation kinetics between the two FPs that can skew the ratio. Even though *in vitro* maturation rates of most FPs are in the tens of minutes, ratios in these experiments frequently drift over hours or even days indicating changes in the relative FP amounts or fluorescence that we do not fully understand. In addition, without knowing the intracellular concentration it is not possible to determine FP brightness on a molecular basis in cells.

Here, we propose an alternative and complementary method to compare the intensity of FPs in mammalian cells by expressing FP-tagged peptides that self-assemble into nanostructures with defined molecule numbers. Because we know how many FP-tagged peptides assemble into one sub-diffraction particle, we can evaluate and compare the brightness of FP variants on a molecule-by-molecule basis in physiologically relevant conditions on the same microscope system that is going to be used for subsequent experiments. This method does not need a reference FP in the same cell and is insensitive to variations in expression level, which only affects the number of particles per cell but not their individual intensity. We use this approach to test which of the recent monomeric StayGold variants performs best in high resolution spinning disc confocal microscopy in mammalian cytoplasm and further compare commonly used red FP variants.

## RESULTS

To quantitatively compare monomeric StayGold variants to each other and to EGFP and mEmerald, as reference standards that are widely utilized in cell biology, we expressed FP-tagged I3-01 peptides in human retinal pigmental epithelial (RPE) cells using the exact sequences of the monomeric StayGold variants as published (Ando et al., 2023; Zhang et al., 2024; Ivorra-Molla et al., 2023). I3-01 is derived from trimeric aldolase (Hsia et al., 2016), and 60 I3-01 subunits self-assemble into stable 26 nm diameter dodecahedron particles in which each of the vertices consists of one I3-01 trimer. These particles are roughly the size of a ribosome and are referred to as I3-01 nanocages. Except for the initial EGFP construct, in which I3-01 is tagged at the C-terminus, we attached all other FPs to the I3-01 N-terminus because recent nanocage structures have indicated that the N-terminus is more clearly oriented toward the nanocage exterior (McCarthy and Gonen, 2022). This is less likely to interfere with nanocage assembly and ensures that FPs of interest are exposed to the cytoplasm. Thus, each nanocage carries 60 FPs (Fig. 1B) allowing comparison of the absolute fluorescence intensity between individual nanocages with different FPs.

We imaged RPE cells expressing green FP-tagged nanocages by spinning disc confocal microscopy, a modality widely used for live-cell imaging, using 488 nm excitation, identical 500 ms exposure times and a 525/50 nm bandpass emission filter that covers the emission peaks of all green FPs tested (Table S1; Fig. S3A). To resolve nanocages as best as possible, we imaged in W1/SoRa super-resolution mode using an excitation irradiance of 15 W cm^−2^ to achieve a good signal-to-noise ratio, especially for dimmer FP variants. Although this is slightly higher than what we would normally use for live-cell time-lapse microscopy to minimize photodamage, this is well within the range of fluorescence and confocal microscopy power densities. Although we were able to detect sub-resolution nanocage particles, most of the fluorescence signal appeared diffusely distributed throughout the cytoplasm (Fig. 1C). Given that nanocages are expected to move freely within the cytoplasm, we hypothesized that this diffuse signal is caused by motion blur of nanocage particles due to the relatively long exposure time rather than non-assembled I3-01 subunits. Indeed, individual fast-moving nanocages were easily observed by total internal reflection (TIRF) microscopy with 50 times shorter exposure times (Fig. 1D; Movie 1). To slow intracellular diffusion and allow capture and analysis of more stationary nanocages by spinning disc confocal microscopy, we therefore increased cytoplasm viscosity by treating cells with 400 mOsm D-mannitol (Shen and Ori-McKenney, 2024). Although this hypertonic treatment did not completely stop nanocage movement, it enabled observation of a sufficiently large number of stationary nanocage particles at longer exposure times, which were most clearly visible in the cell periphery with nearly no cytoplasm background fluorescence, indicating highly efficient nanocage assembly and long-term stability (Fig. 1E,F; Fig. S1).

To quantitatively compare the brightness of different FP-tagged nanocages, we fitted 2D Gaussian distributions to individual sub-resolution nanocage particles corresponding to 60 FP molecules each and, removing the local background as the offset of the 2D Gaussian (Fig. 1F), integrated the fluorescence intensity inside a circle with a radius of two standard deviations. This direct comparison of nanocage particle intensities showed that the average intensity of all monomeric StayGold variant nanocages was approximately three times higher than EGFP or mEmerald (Fig. 1G), which despite being linked to I3-01 at opposite ends were not significantly different from one another. Because the excitation peak of all StayGold variants is red-shifted and, compared with EGFP and mEmerald, are less efficiently excited at 488 nm, the real brightness increase compared to EGFP and mEmerald might be even greater (Table S1). mStayGold nanocages also appeared to be slightly brighter than mBaoJin, but this difference was not statistically significant. Although the total intensity of mStayGold-expressing cells was higher than of EGFP-expressing cells, given the higher intensity of mStayGold nanocages, the total number of nanocage particles per cell was similar (Fig. 1H). Of note, although a range of expression levels is expected in transient transfections, even in the brightest cells analyzed, the number of nanocages was orders of magnitude below the estimated number of ribosomes in a typical mammalian cell, indicating reasonably low nanocage expression levels in our analyzed cell population. The intensity standard deviation of FP-tagged nanocage populations scaled linearly with the mean nanocage intensity (Fig. S1B) indicating similar relative variability that is thus independent of the specific FP tag, with the notable exception of StayGold(E138D). The increased intensity variability of StayGold(E138D) nanocages likely indicates aggregation (Fig. 1F; Fig. S1). Although this might lead to an overestimation of nanocage intensity by our method, StayGold(E138D) was also poorly expressed (Fig. 1H), and we conclude that StayGold(E138D) is a poor choice for mammalian cells.

FPs are connected to the outside of nanocage particles by 2–3 nm long GGS linkers. Given the length of the FP molecules (∼4 nm), the centers of nanocage-linked FP molecules are distributed on the surface of a sphere with a diameter of ∼35 nm. Using a Fibonacci lattice to distribute 60 points evenly on a sphere (González, 2010), this results in an average spacing of 7–8 nm between FPs, which is larger than the <5 nm distances at which noticeable fluorophore self-quenching is observed (Meredith et al., 2024). Although it is thus unlikely that self-quenching impacted our measurements to a significant degree, we compared the fluorescence intensities of mEmerald and mStayGold nanocages tagged with either one or two FP tags in tandem with a short linker between the two. In contrast to mEmerald nanocages, for which the fluorescence intensity increased linearly, nanocages with 120 mStayGold molecules were ∼10% less bright than expected (Fig. 1I). This might indicate that mStayGold is more prone to fluorescence quenching at high local concentrations, which should be considered if nanocages are used as microscopy fluorescence standards (Akamatsu et al., 2020; Dema et al., 2024).

**Fig. 2.**
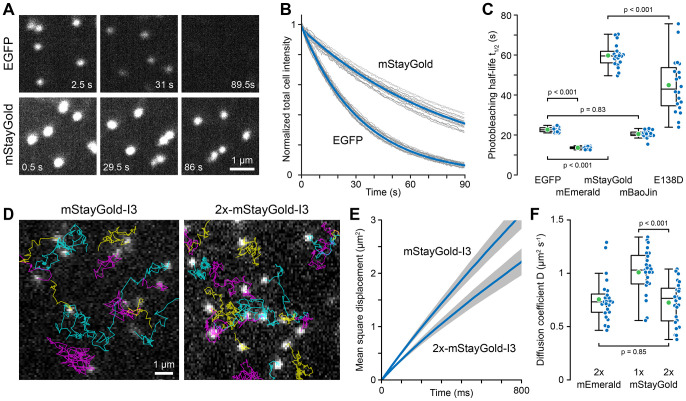
**Green FP-tagged I3-01 nanocage photostability and intracellular diffusion.** (A) Representative EGFP and mStayGold nanocage photobleaching images. Images are scaled to the same absolute intensities. (B) Photobleaching as a function of exposure time. Gray curves are measurements from individual cells normalized to the fluorescence intensity at the beginning of the time series. Blue curves are single exponential fits of all data. (C) Comparison of photobleaching half-lives. *n*=20 cells per condition. (D) Representative TIRF microscopy images from 85 frames per second time series to measure nanocage diffusion. Particle tracks are overlaid in color. (E) Average mean square displacement curves from 25 cells per condition representing a total of 16,504 mStayGold (1×) and 20,692 tandem-tagged mStayGold–mStayGold (2×) nanocage particle tracks. Gray areas indicate 95% confidence intervals. (F) Comparison of the diffusion coefficients of the indicated nanocage constructs. *n*=25 cells per condition. Of note, single-tagged (1×) mEmerald nanocages were too dim to track adequately. In C and F, green circles indicate the mean, blue symbols are data from individual cells. Box plots show median, first and third quartile, with whiskers extending to observations within 1.5 times IQR from the quartile 1 and 3 boundaries. Statistical analysis by one-way ANOVA with Tukey–Kramer honestly significant difference (HSD) test.

Photostability is the second crucial parameter for live microscopy experiments. As expected, photobleaching kinetics were identical for individual nanocages or whole cells (Fig. S2A). For the ease of measurement, we therefore compared the photobleaching rate by measuring the decay in whole-cell fluorescence under continuous 15 W cm^−2^ illumination (Fig. 2A–C; Movie 2). Photobleaching of all green FPs was captured adequately by single exponential decay curve fitting (Fig. S2B), and comparing fluorescence half-lives, we found that mStayGold was nearly three times as photostable (t_1/2_=60±5.6 s; mean±s.d.) as EGFP (t_1/2_=23±1.2 s). To our surprise, however, mBaoJin photobleached rapidly (t_1/2_=20±1.8 s) with kinetics indistinguishable from EGFP.

Although all FP-tagged nanocages appeared as sub-diffraction objects with a full width at half maximum (FWHM) near the resolution limit of our spinning disc microscope system (mEmerald, 202±20 nm; mStayGold, 208±20 nm, mean±s.d.), to ascertain that the increased brightness of mStayGold nanocages was not due to aggregation, we compared intracellular diffusion of mEmerald and mStayGold-tagged nanocages. Nanocage movement was tracked in image sequences acquired by TIRF microscopy at 85 frames per second (Fig. 2D) and apparent diffusion coefficients determined by mean square displacement over time assuming non-directional Brownian motion (Fig. 2E). Nanocages with one mStayGold molecule per I3-01 peptide had an apparent diffusion coefficient of *D*=1.01±0.21 µm^2^ s^−1^ (mean±s.d.) whereas the diffusion coefficient of tandem-tagged mStayGold–mStayGold nanocages was reduced to 0.72±0.20 µm^2^ s^−1^. At low Reynolds number, the diffusion coefficient is approximately inversely linearly related to particle size and this ∼25% decrease in diffusion coefficient is consistent with an ∼25% increase in effective nanocage size due to addition of another layer of mStayGold molecules to the nanocage exterior, increasing the diameter by ∼8 nm. Importantly, the diffusion coefficient of mEmerald–mEmerald nanocages (*D*=0.76±0.20 µm^2^) was indistinguishable from mStayGold–mStayGold nanocages indicating that these particles have the same size and therefore the increased brightness of mStayGold nanocages is not caused by aggregation (Fig. 2F).

**Fig. 3.**
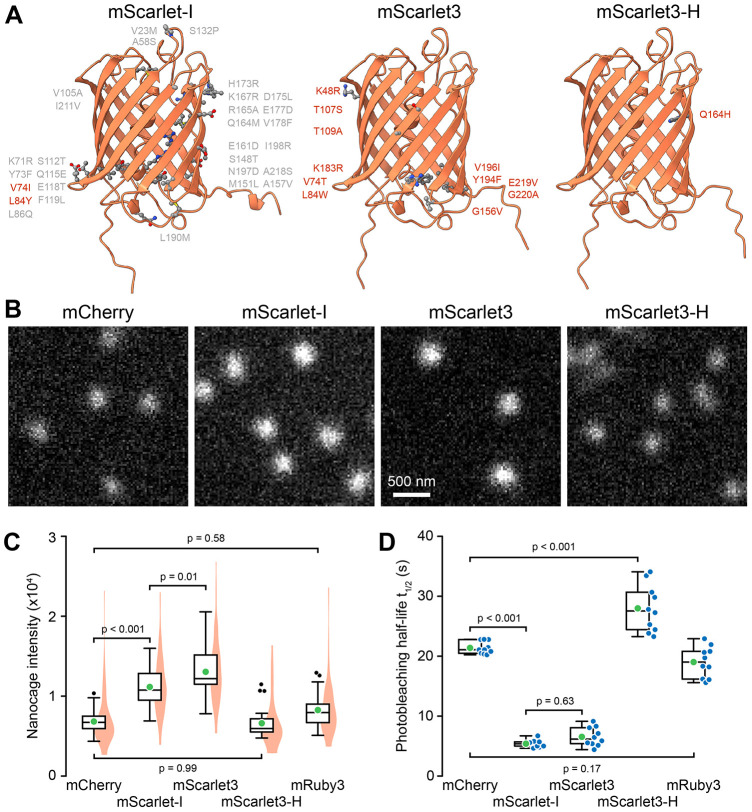
**Red FP-tagged I3-01 nanocage fluorescence intensity and photostability.** (A) AlphaFold2 structures of the three mScarlet variants tested. Compared to mCherry, all mScarlet variants share the amino acid substitutions labeled in gray. Amino acid substitutions highlighted in red are specific to the different mScarlet variants. (B) Comparable peripheral cell regions of D-mannitol-treated RPE cells expressing the indicated red FP nanocage fusions. Images were acquired with the same exposure settings and are scaled to the same absolute intensities. (C) Comparison of the integrated intensity of nanocage particles tagged with the indicated FP. *n*=30 cells each from three independent experiments. Violin plots show the intensity distribution of all nanocage particles with 10 nanocages analyzed per cell. (D) Comparison of photobleaching half-lives. *n*=10 cells per condition. In C and D, green circles indicate the mean, blue symbols are data from individual cells. Box plots show median, first and third quartile, with whiskers extending to observations within 1.5 times IQR from the quartile 1 and 3 boundaries. Statistical analysis by one-way ANOVA with Tukey–Kramer honestly significant difference (HSD) test.

To test whether our nanocage approach works for benchmarking other wavelengths FPs and because the choice of available red FPs is even more confusing, we next compared mCherry, one of the first optimized red FPs from *Discosoma* (Shaner et al., 2004), to more recently developed mCherry-derived mScarlet variants (Fig. 3A). Compared with mCherry, both mScarlet-I- (Bindels et al., 2017) and mScarlet3-tagged (Gadella et al., 2023) nanocages were 1.6- and 1.9-fold brighter, respectively (Fig. 3B,C). This increase in nanocage intensity was surprisingly smaller than that previously reported using ratiometric imaging (Gadella et al., 2023). A difference in raw data can be explained by differences in imaging systems. Whereas we used a long-pass filter to gather as much of the red FP emission spectra as possible (Table S1; Fig. S3B), Gadella et al. employed a band-pass filter that better matches the blue-shifted mScarlet emission spectrum as well as a shorter excitation wavelength. However, this difference remained after corrections for the spectroscopic throughput of either imaging system (Table S1).

In addition, this moderate increase in brightness was eclipsed by a dramatic nearly 4-fold decrease in photostability of both mScarlet-I (t_1/2_=5.4±0.6 s) and mScarlet3 (t_1/2_=6.5±1.6 s) compared with mCherry (t_1/2_=21±1.0 s) (Fig. 3D). Recently, other mScarlet variants with improved photostability have been developed. We tested one of those, mScarlet3-H, in which glutamine 164 of mScarlet3 is changed into a histidine residue (Fig. 3A) (Xiong et al., 2025). Although, this single amino acid substitution substantially reduced photobleaching, it also reduced brightness to a level indistinguishable to mCherry. Finally, we tested mRuby3, an optimized red FP derived from a different anthozoan, *Entacmaea quadricolor* (Bajar et al., 2016), which was indistinguishable from mCherry in both brightness and photobleaching kinetics (Fig. 3C,D).

## DISCUSSION

**Fig. 4.**
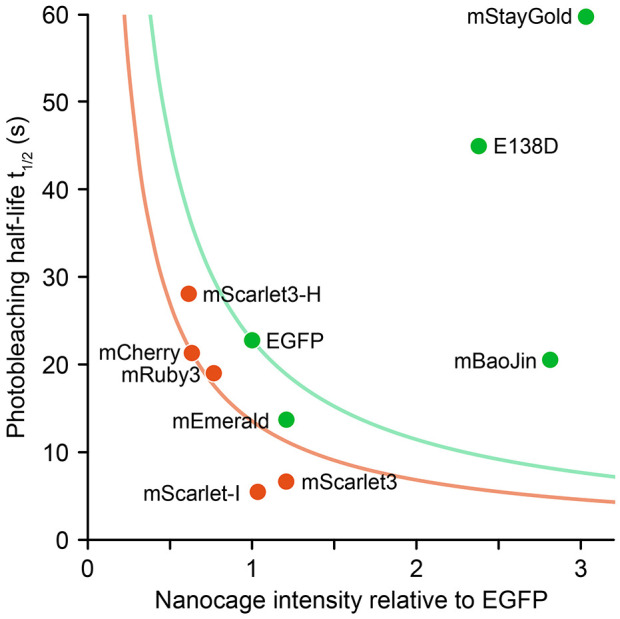
**Comparison of brightness relative to EGFP and photobleaching half-life of all FPs tested.** To compare FP performance in live spinning disc confocal microscopy experiments we defined a relative emission yield metric as the product of relative brightness and photobleaching half-life at the same irradiance (Table S1). The hyperbolic curves through EGFP and mCherry thus indicate FPs with the same relative emission yield that would be expected to behave similarly if imaged at the same starting emission signal. FPs to the right and above these curves are expected to perform better in live microscopy, with mStayGold all the way in the upper right corner of the plot. Surprisingly, none of the tested red FPs performed substantially better than mCherry. Of note, this is specific to the imaging setup used and will look different with different excitation and emission channels that influence both apparent intensity and photobleaching kinetics.

We present a new method to compare the brightness of different FPs on a molecule-by-molecule basis by quantifying the intensity of individual FP-tagged nanocages in live cells. We believe this method complements existing ratiometric approaches but has notable advantages. By slowing intracellular nanocage diffusion, it can be adopted to other imaging modalities or cell types and is independent of relative expression levels or ratio measurements, thus providing a straightforward assay to test which FP might be best for a specific experimental setup, as nanocage intensity and photobleaching measurements can be performed on the same set of transfected cells. For live spinning disc confocal microscopy with typical excitation and emission channels, we find that the original mStayGold (Ando et al., 2023) far outperformed older green FPs and other monomeric StayGold variants. Although mStayGold and mBaoJin were both much brighter than EGFP, mStayGold was exceptionally photostable, and combining our measurements of increased nanocage intensity and increased photostability, we conclude that the original mStayGold (Ando et al., 2023) is far superior to traditional GFPs, allowing up to ten times as many exposures as EGFP or mEmerald with a similar fluorescence signal at reduced excitation intensity (Table S1; Fig. 4). This conclusion independently supports similar findings from a comparison of the three monomeric StayGold variants using the ratiometric approach by the Miyawaki laboratory who both initially isolated StayGold and developed the original monomeric mStayGold (Shimozono et al., 2024 preprint). Because performance in live cells was our primary objective, we did not further investigate StayGold(E138D) aggregation or poor expression, although a contributing factor might be that StayGold(E138D) is not codon-optimized for mammalian expression (Ivorra-Molla et al., 2023).

We also show that mStayGold nanocages move freely in the cytoplasm with diffusion coefficients similar to values reported for cytoplasmic diffusion of similar sized nanoparticles (Delarue et al., 2018; Molines et al., 2022), demonstrating that mStayGold nanocages can serve as improved monodisperse and highly photostable nanoparticles for the biophysical characterization of intracellular environments that can be tracked with high fidelity for hundreds of frames. Because of their defined brightness and photostability, in lieu of fluorescent beads, mStayGold nanocages may also better represent biological specimens for standardized comparison of microscope system characteristics.

Our limited comparison of red FPs was more challenging as their emission spectra are more diverse, and therefore relative intensity comparisons are complicated by the exact fluorescence channels used. However, even after correction for imaging system spectral differences, both mScarlet-I and mScarlet3 were surprisingly inferior to mCherry in our spinning disc live microscopy setting (Table S1; Fig. 4) mostly due to very rapid photobleaching. The mechanisms that determine FP photostability remain poorly understood, but there appears to be a strong inverse relationship between brightness and photostability in these mCherry-derived FPs that may be related to the rigidity of the chromophore environment, consistent with newer more photostable but dimmer mScarlet3 variants (Xu et al., 2024 preprint; Xiong et al., 2025). It is unclear why we find that mScarlet variants are less bright than previously reported (Gadella et al., 2023). One possibility is that FP spectra in cells are different from reported spectra *in vitro* (Lambert, 2019), which would skew spectral corrections. Alternatively, photobleaching may be non-linearly related to the excitation wavelength or power. In any case, for practical purposes, this highlights the importance of independently evaluating FP performance on the imaging system that is going to be used for a specific experiment, and of adopting a standard to compare FPs in identical and experimentally relevant conditions. We propose that FP-tagged I3-01 nanocages could become such a standard. Because we used the same irradiance for both green and red FPs, we can also directly compare the relative brightness of different color FPs showing that in general all red FPs were less bright than EGFP, further highlighting how much mStayGold is set apart from all the FPs that have been described over the last decades (Fig. 4), and the need for other wavelength FPs with similar performance.

## MATERIALS AND METHODS

### Molecular cloning

Mammalian codon optimized I3-01 K129A (Akamatsu et al., 2020) was cloned by Gibson assembly into a BamHI-digested pEGFP-N1 plasmid (Clontech) to generate a plasmid expressing I3-01–EGFP. Plasmids expressing mEmerald–I3-01 were as described previously (Dema et al., 2023). All other I3-01 nanocage constructs tagged either with a single or a tandem repeat of the indicated fluorescent proteins at the N-terminus were constructed by Gibson assembly with four to seven GGS spacers between the FP and the I3-01 nanocage peptide. The mStayGold coding sequence was PCR amplified from pCMV3-3xNLS-mStayGold (a gift from Cuyler Luck, University of California, San Francisco, USA). The mBaoJin coding sequence was synthesized as a gene fragment (TWIST Biosciences) based on the sequence reported in Zhang et al. (2024). The E138D coding sequence was PCR amplified from pcDNA3-10His-mStayGold(E138D) [Addgene plasmid #211363; deposited by Mohan Balasubramanian (RRID:Addgene_211363)]. All red FP constructs were synthesized as gene fragments and cloned into the mStayGold-I3-01 backbone after removing the mStayGold coding sequence by BamHI restriction digest. All constructs were verified by whole plasmid sequencing (Primordium Labs). Protein structural models were computed using the AlphaFold2 Google Colab (Mirdita et al., 2022) using the protein sequences as input. All I3-01 FP plasmids will be made available in Addgene.

### Cell culture and live microscopy

RPE cells were cultured in DMEM/F12 medium (Invitrogen) supplemented with 10% fetal bovine serum (Atlanta Biosciences) and 2 mM glutamine (Invitrogen) at 37°C and 5% CO_2_ (Webb et al., 2021). For transient nanocage expression and microscopy, cells were plated in 35-mm #1.5 glass-bottom dishes (MatTek), and transfected after 24 h using jetOPTIMUS (Sartorius), and used for experiments 24 h later. To increase extracellular osmolarity, the tissue culture medium was exchanged with medium supplemented with 0.28 mM (i.e. 400 mOsm) D-mannitol (M4125; Sigma-Aldrich) 10–15 min before imaging.

High-resolution spinning disc image sequences of nanocage-expressing RPE cells were acquired with a CFI Apochromat TIRF 60× NA 1.49 CFI Apochromat objective (Nikon) on a Yokogawa CSU-W1/SoRa spinning disc confocal system and an ORCA Fusion BT sCMOS camera (Hamamatsu) controlled through NIS Elements v5.3 software (Nikon) yielding an effective pixel size of 27 nm. All spinning disc experiments used the same 500 ms exposure with an irradiance of ∼15 W cm^−2^ to achieve a high signal-to-noise ratio and ensure consistency between experiments. For green FPs, 488 nm excitation and a bandpass emission filter with a center wavelength at 525 nm (Semrock FF03-525/50) were used, a common configuration for GFP imaging. For red FPs, we used 561 nm excitation and a long-pass emission filter with a 50% edge at 579 nm (Semrock BLP01-568R) to cover as much as possible of the more diverse red FP emission spectra.

TIRF image sequences were acquired on a TI inverted microscope (Nikon) equipped with a motorized TIRF illuminator (Nikon) with a 100×1.49 NA CFI Apochromat TIRF objective (Nikon) using 1.5× intermediate magnification and an iXon EMCCD camera (Andor) yielding an effective pixel size of 110 nm. The camera region of interest (ROI) was set to 150×512 pixels to achieve 85 frames per second acquisition rates.

### Image analysis

Fluorescence intensities of different FP-tagged nanocages were compared from the same imaging session to minimize day-to-day instrument variability. Nanocages were analyzed in the first to third image of short *Z*-stacks to select the plane with optimal focus but also minimize the impact of photobleaching on our data. The integrated fluorescence intensity of individual nanocages was quantified with custom-written MATLAB code, NanoQuant.m (https://github.com/TorstenWittmann/NanoQuant), to fit 2D Gaussian functions to user-selected nanocage particles. ChatGPT was used for help with coding. Fluorescence intensity from the original image data inside a circle with a radius of two standard deviations, which accounts for ∼86% of the total volume of a 2D Gaussian distribution (Wang et al., 2015), around the maximum of the Gaussian fit minus the offset of the fit representing the local background was integrated to calculate the nanocage fluorescence signal. To analyze photobleaching, the average intensity in an ROI encompassing the entire cell was measured over time and each cell photobleaching curve fitted with an exponential decay function to determine photobleaching half-life.

Nanocage particles in TIRF image sequences were tracked in MATLAB using the u-track package (https://github.com/DanuserLab/u-track; Jaqaman et al., 2008) with Gaussian mixture model detection and default settings. Individual nanocage particle tracks longer than 20 frames and shorter than 250 frames (i.e. <3 s) were extracted from the ‘tracksFinal’ structure, and diffusion coefficients determined by linear fitting of the initial 25% of the average mean square displacement curve per cell using the @msdanalyzer package (https://tinevez.github.io/msdanalyzer/; Tarantino et al., 2014) assuming Brownian motion at short time scales.

### Statistics

Details of statistical analysis including the type of test, *P*-values and numbers of biological replicates are provided within the relevant figures and figure legends. All statistical analysis was done in MATLAB (Mathworks, Inc.), graphs were produced in MATLAB and in Excel (Microsoft), and figures assembled with Adobe Illustrator. In all figures, box plots show median, first and third quartile, with whiskers extending to observations within 1.5 times the interquartile range (IQR) from the quartile 1 and 3 boundaries.

## Supplementary Material



10.1242/joces.263858_sup1 Supplementary information
