# Supplementary material for: Quantitative comparison of fluorescent proteins using protein nanocages in live cells
Source: J Cell Sci. 2025 May 21;138(10):jcs263858. doi: 10.1242/jcs.263858 (PMC12148025; doi:10.1242/jcs.263858)
Supplement: Supplementary information [file joces-138-263858-s1.pdf]

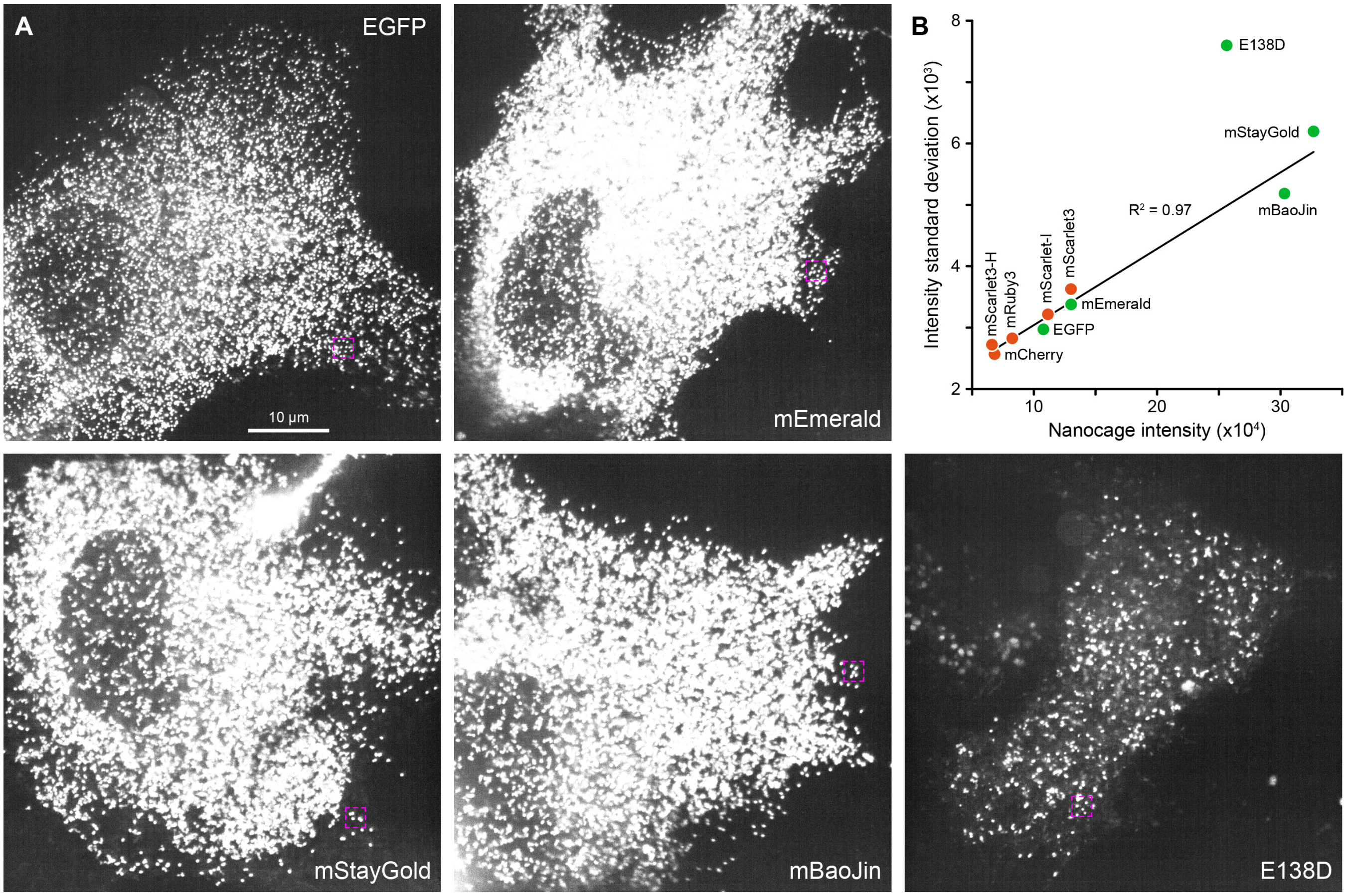

Fig. S1. Green FP nanocage-expressing cells. (A) Example images of D-Mannitol-treated RPE cells expressing I3-01 nanocages tagged with the indicated green FPs. Regions shown in Fig. 1F are indicated, but images are scaled to a higher contrast by lowering the upper intensity threshold to illustrate that there is very little fluorescence in the cytoplasm from potentially unassembled I3-01 peptide. Of note, this scaling saturates the nanocage particle signal and does not allow a proper brightness comparison. Individual nanocages are clearly visible in the cell periphery and underneath the nucleus but are too dense to be resolved in the cell interior. (B) With the exception of E138D, the nanocage intensity standard deviation scales linearly with nanocage intensity, which indicates a similar relative variability that is independent of the FP tag.

Journal of Cell Science • Supplementary information

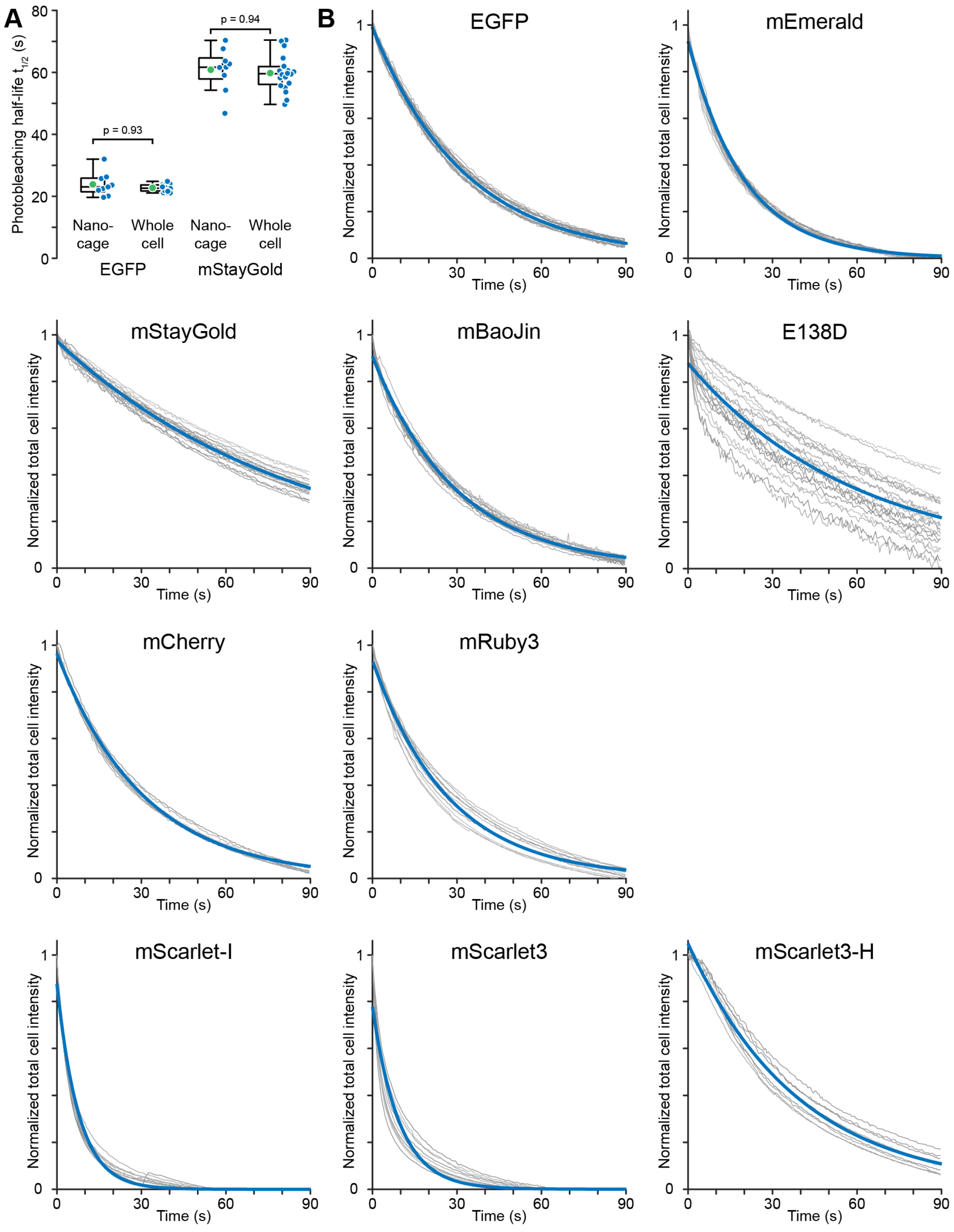

Fig. S2. Photobleaching kinetics. (A) Comparison of photobleaching half-lives of EGFP and mStayGold from measurements of the integrated intensity of individual nanocages (from n = 9 cells) or from whole cells (same data as in Fig. 2C). (B) All normalized whole cell photobleaching curves used to determine photobleaching half-lives. Grey lines are data from individual cells. Blue lines are exponential fits of the average. Note that mScarlet photobleaching is poorly described by a single exponential but the fit is still useful to estimate the photobleaching half-life.

Journal of Cell Science • Supplementary information

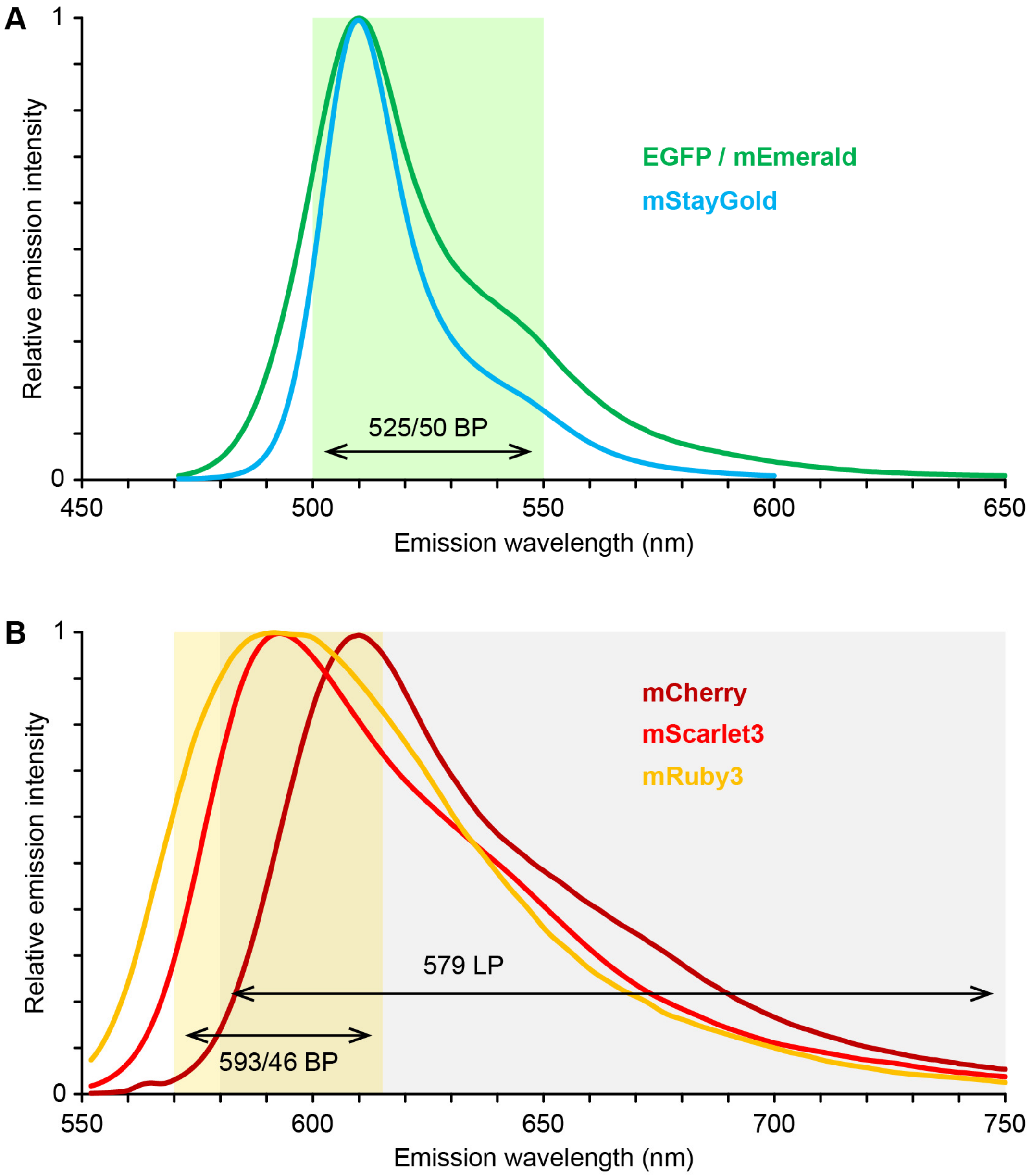

Fig. S3. Emission spectra. (A) Comparison of the emission spectra of EGFP and mStayGold showing the emission bandpass filter used here. (B) Comparison of the emission spectra of mCherry, mScarlet3 and mRuby3 showing the longpass (579 LP) filter used here (grey) as well as the bandpass filter used by Gadella et al. (yellow) that is sub-optimal for collecting mCherry emission. All spectra are from www.fpbase.org (Lambert, 2019).

Journal of Cell Science • Supplementary information

Table S1. Comparison of relative brightness differences for all tested FPs.

	Nanocage intensity relative to EGFP[1]	Photobleaching $t_{1/2}$ (s)[2]	Relative emission yield[3]	Excitation efficiency relative to peak λ_{Ex}	Fraction of emission spectrum recorded	Corrected intensity relative to EGFP[4]
				$\epsilon_{488}/\epsilon_{max}$	with 525/50 BP	
**EGFP**	1.0 ± 0.16	22.7 ± 1.20	1.0	1 [488 nm]	0.72	1.0
**mEmerald**	1.2 ± 0.19	13.6 ± 0.63	0.7	1 [488 nm]	0.72	1.2
**mStaygold**	3.0 ± 0.33	59.8 ± 5.63	8.0	0.59 [500 nm]	0.85	4.4
**mBaojin**	2.8 ± 0.27	20.5 ± 1.76	2.5	0.62 [500 nm]	0.78	4.2
**E138D**	2.4 ± 0.53	45.0 ± 13.84	4.7	0.62 [497 nm]	0.8	3.5
				$\epsilon_{561}/\epsilon_{max}$	with 579 LP	
**mCherry**	0.6 ± 0.12	21.3 ± 1.04	0.6	0.64 [587 nm]	0.99	0.7
**mScarlet-I**	1.0 ± 0.21	5.4 ± 0.60	0.2	0.82 [569 nm]	0.91	1.0
**mScarlet3**	1.2 ± 0.26	6.5 ± 1.56	0.3	0.87 [569 nm]	0.92	1.1
**mScarlet3-H**	0.6 ± 0.16	28.0 ± 3.91	0.8	0.86 [551 nm]	0.93	0.6
**mRuby3**	0.8 ± 0.18	18.9 ± 2.59	0.6	0.97 [558 nm]	0.85	0.7

Notes:

[1] Mean ± standard deviation of measurements from n = 30 cells (from three independent experiments and ten nanocage particless measured per cell).

[2] Mean ± standard deviation of measurements from n = 20 (green FPs) and n = 10 (red FPs) cells (from three independent experiments).

[3] Assuming a linear relationship between irradiance and photobleaching rate ($k_{bleach} \propto E$) at below saturation illumination levels and single-photon absorption, we define the relative emission yield (Y) as the difference in the FP functional lifetime at the same initial emission signal (I) relative to EGFP as a reference, estimated as:

$$Y = (I \times t_{1/2}) / (I_{EGFP} \times t_{1/2,\ EGFP})$$

Thus, in a time-lapse experiment with the same initial signal at t_0 (i.e. 3-fold lower excitation intensity at 488 nm), mStayGold is expected to last 8-times as long as EGFP.

Journal of Cell Science • Supplementary information

[4] Difference in relative nanocage intensity normalized for the difference in excitation efficiency (ϵ) at 488 nm or 561 nm, respectively, and the fraction (f) of the integrated emission spectrum collected relative to EGFP:

$$I_{corr} = I \times {}^{\epsilon_{max}}/_{\epsilon_{488/561}} \times {}^{f_{Em,EGFP}}/_{f_{Em,FP}}$$

This estimates the real difference in nanocage intensity if the FP in question were excited at the peak excitation wavelength and the integrated emission over the whole spectrum were collected. Of note, this does not take into account other spectral differences of the imaging system such as for example the quantum efficiency spectrum of the camera or differences of the FP spectra in the intracellular environment and thus has to remain an estimate.

Journal of Cell Science • Supplementary information

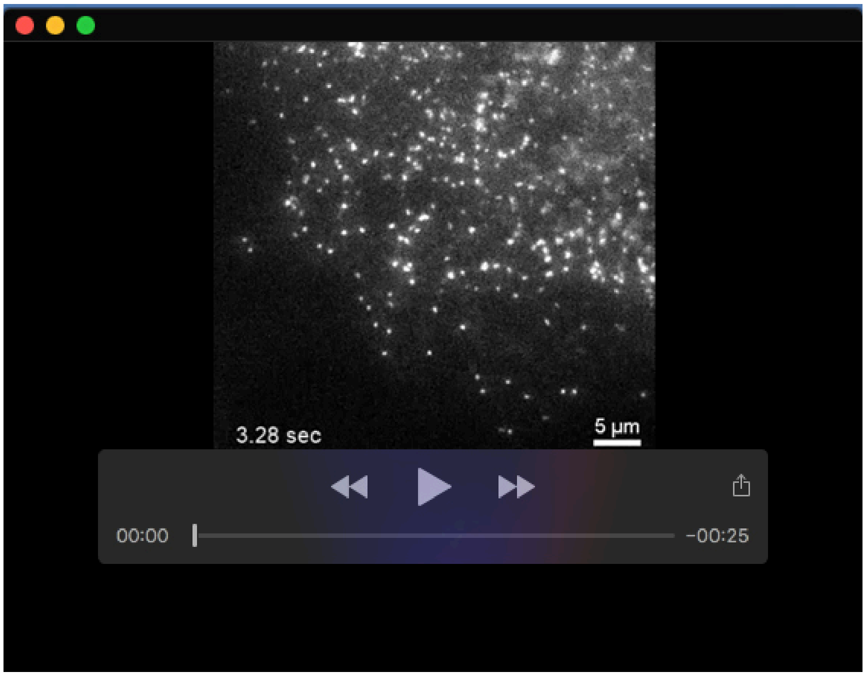

Movie 1. mStayGold I3-01 nanocage diffusion by TIRF microscopy in a transfected RPE cell. Acquisition at 22 frames s^{-1}.

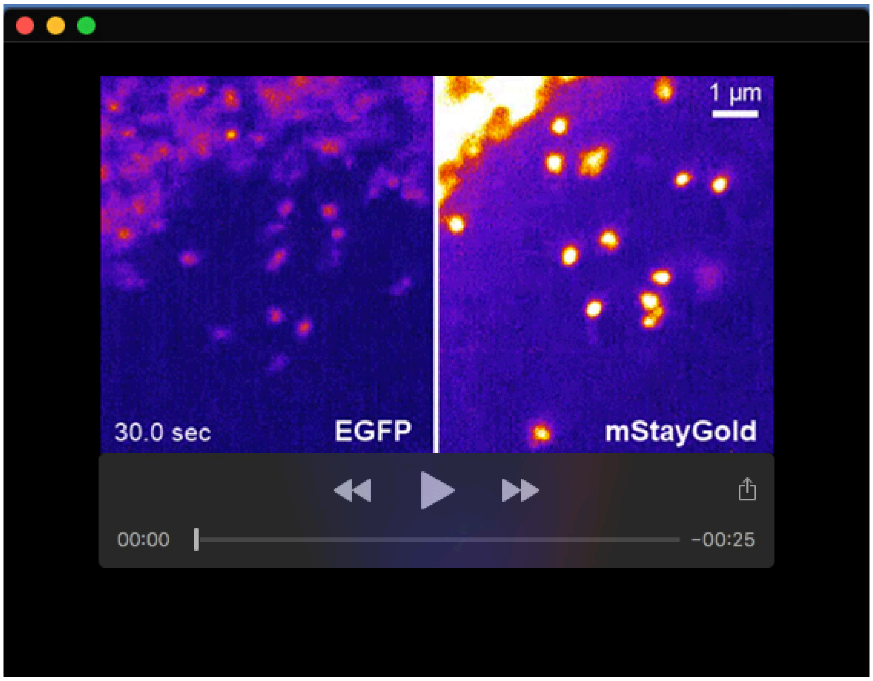

Movie 2. Photobleaching comparison of EGFP and mStayGold I3-01 nanocages in 400 mOsm D-mannitol-treated RPE cells by spinning disk confocal microscopy under continuous 15 W cm^{-2} illumination. Images are scaled to the same absolute intensities and shown in pseudocolor. Note that the mStayGold nanocages at the end of the video are still brighter than the EGFP nanocages in the beginning. Acquisition at 2 frames s^{-1}.

Journal of Cell Science • Supplementary information
