# Supplementary material for: Quantitative comparison of fluorescent proteins using protein nanocages in live cells
Source: J Cell Sci. 2025 May 21;138(10):jcs263858. doi: 10.1242/jcs.263858 (PMC12148025; doi:10.1242/jcs.263858)
Supplement: Supplementary file 1 [file joces-138-263858_review_history.pdf]

Journal of Cell Science

Quantitative comparison of fluorescent proteins using protein nanocages in live cells

Giulia Viola, Yasmeen W. Ibrahim, Kyle A. Jacobs, Joël Lemière, Matthew L. Kutys and Torsten Wittmann
DOI: 10.1242/jcs.263858

Editor: Michael Way

Review timeline

Original submission:	16 January 2025
Editorial decision:	24 February 2025
Editorial decision:	9 April 2025
First revision received:	24 April 2025
Accepted:	25 April 2025

Original submission

First decision letter

MS ID#: jcs.263858

MS TITLE: Quantitative Comparison of Monomeric StayGold Variants Using Protein Nanocages in Living Cells

AUTHORS: Giulia Viola; Yasmeen Ibrahim; Kyle A Jacobs; Joel Lemiere; Matthew L Kutys; Torsten Wittmann
ARTICLE TYPE: Tools and Resources

Dear Torsten,

Sorry its taken longer than I would like but we have now reached a decision on the above manuscript.

To see the reviewers' reports and a copy of this decision letter, please go to:

As you will see from their reports, the reviewers raise a number of substantial criticisms that I'm afraid prevent me from inviting revision given the level of criticism especially from reviewer 2..

I am very sorry to give you such disappointing news, after encouraging you to submit your paper to JCS but it requires a largely positive recommendation by the referees for a manuscript to progress.

I do hope you find the comments of the reviewers helpful in allowing you to revise the manuscript for submission elsewhere, and many thanks for sending your work to Journal of Cell Science.

Reviewer 1

In this article, Giulia Viola et al. introduce a new strategy for comparing FP properties in cells using Nanocages. Using this method, they compare several FPs, including mStayGold, mBaoJin, and StayGold(E138D), and several mScarlet variants. The method proposed is interesting, but several questions remain before I can recommend publication.

© 2025. Published by The Company of Biologists under the terms of the Creative Commons Attribution License (https://creativecommons.org/licenses/by/4.0/).

Major comments:
The manuscript currently does not sufficiently acknowledge or contrast the presented methodology with existing approaches for assessing FP properties in live cells. For example, ratiometric analyses involving co-expression of FPs like mCherry to normalize expression levels are standard and should be discussed explicitly in the introduction. The authors should clarify why their approach is superior or complementary to such methods. While the presented method is innovative, it would benefit from a discussion on scenarios where it may outperform or be less suitable than other methods.

A critical question arises regarding the efficiency of nanocage assembly. How can the authors ensure that the nanocages are complete in cells? Presumably, the FP will fluoresce regardless of the number of subunits in the cage. Is the assembly uniform across the different FPs tested? The variability observed in StayGold(E138D) fluorescence intensity (Fig. 1F) suggests incomplete assembly or aggregation, which warrants further investigation. Please include quantitative data on the efficiency of the cage assembly and whether it varies with FP choice.

It is also unclear how having sixty FPs in close proximity (in the cage) impacts the fluorescence properties of individual FPs. Could proximity-based quenching, Förster resonance energy transfer, or photophysical effects alter brightness or photostability?

One major limitation of testing fluorescence properties in cells is that it does not always recapitulate the in vivo situation in tissues or whole organisms. How easily can the nanocage method be adapted for use in model organisms? Addressing the feasibility of this approach for in vivo studies would greatly broaden its impact.

Minor Comments:
The title does not reflect the study as other FP than StayGold are tested.

Reviewer 2

Giulia et al report on the comparison of monomeric StayGold variants in living cells. To this end they expressed fusion proteins that self-assemble into nanocages and they studied the brightness and photostability using spinning disk confocal microscopy. They report that mStayGold outperforms EGFP and is better than the E138D and mBaoJin monomeric versions of mStayGold.

1) Relevant papers are not cited that describe much more elaborate quantitative comparison of the 3 monomeric StayGold variants in cells which was published one year ago (Shimozono et al. (2024) BioRxiv, doi: 10.1101/2024.09.16.613379). Hence, their statement on page 3 "Although it is critical to select the brightest and most photostable fluorescent protein for live microscopy, no independent side-by-side comparison of these variants has been made" is misleading. Also not cited is the paper by Hirano et al (2024) Sci. Rep. 14:5541 which very elaborately descibes the behavior of StayGold under various illumination conditions in different microscopy implementations and show that the photostability is very dependent on the exact illumination conditions.
Note that both the above papers were published before the current study appeared online (February/March 2024 versus September 2024).
2) The findings on mStaygold variants in essence confirm earlier published data: mStayGold is better than mBaoJin (equally bright but more photostable) and E138D (equally photostable but less bright), see Shimozono et al. (2024) BioRxiv, doi: 10.1101/2024.09.16.613379). So the overall novelty of the findings is questionable.
3) mStayGold was reported to be sensitive towards tagging at the N-terminus (Ando et al, 2023). No sequence information is available about the fusion constructs used in the current study. For instance, the brightness of E138D can be greatly affected by small sequence additions at the N-terminus (Shimozono et al. (2024)). So likely the fusion to the N-terminus of the nanocage forming peptides will affect the bightness of some of the tested variants.
4) The authors claim that the FP- nanocages allow quantitative comparison. Yet here it is assumed that a) the cages bringing many FPs in close proximity in an artificial environment do not change the properties of these FPs (for which no evidence is provided), b) that all tested FPs have no residual tendency for oligomerization, influencing the size/number of FPs per nanocage (no evidence provided). In this respect I cite the authors on page 5, line 1: "StayGold(E138D) nanocage

© 2025. Published by The Company of Biologists under the terms of the Creative Commons Attribution License (https://creativecommons.org/licenses/by/4.0/).

particle population was highly variable indicating either incomplete nanocage assembly or aggregation (Fig. 1F)". So here the quantitative brightness comparison is flawed as a result of this approach. Note for instance, that EGFP is a dimer since it contains an Alanine206.

5) The authors misleadingly state that "it is difficult to quantitatively compare FP characteristics in live cells" (page 5, line 42) and that these nanocages allow "compare the intrinsic brightness of FP variants on a molecule-by-molecule basis in physiologically relevant conditions" (page 1 line 56). For cell biologists these nanocages are irrelevant, since they are truly artificial non-physiological large assemblies within the cytoplasm. Furthermore, in this study even no corrections are performed for expression level differences. They state that "a large variability is expected in transient transfections" (page 5, line 8) yet they continue to compare single transfected cells. It is of note that in the study of Shimozono (2024) quantitative co-expression was done using 2A sequences that allow 1:1 production of diverse green FPs togetgher with a red FP. Also elaborate brightness comparisons were done for all RFPs in which careful expression corrections and (in addition) spectral corrections accounting for different spectroscopic features were done both in living and in fixed cells (Gadella et al. 2023). Note that such data show that in cells mScarlet3 is >5 times brighter and not 2 times brighter than mCherry as is suggested in Supplemental Table I. These discrepancies likely reflect differences in transfection efficiency which are not corrected for.

6) No corrections are done for spectroscopic throughput. Despite differences in excitation/emission spectra in EGFP, mEmerald and the three mStayGolds. So the numbers obtained are biased by the employed excitation lines and emission filters in the microscopy setup.

7) The illumination power used for all experiments (15 W/cm2), page 19, line 42, is abnormally high and not in any way relevant for regular cell biological fluorescence microscopy experiments.

8) The maturation of these FPs in cells is a characteristic of major importance and relevance for cell biologists but completely neglected in the current study. Both the speed and final extent of chromophore maturation are critical parameters for comparing FPs. While these parameters are tested extensively for these FPs in other studies (see Ando et al., 2023, Shimozono et al. (2024) Gadella et al (2023), no information is found on this in the current comparison.

Considering the above I cannot recommend publication, even not after major revision.

First revision

<u>Author response to reviewers' comments</u>

Response to Reviewers:

We thank the reviewers for their insightful comments. Altogether, this has made us dive deeper on several of the criticisms raised and believe ultimately have made this a better manuscript. We provide detailed point-by-point responses to all the points raised by the reviewers below. Substantial changes in the text are highlighted in blue.

Comments from the Reviewers:

Reviewer 1:

In this article, Giulia Viola et al. introduce a new strategy for comparing FP properties in cells using Nanocages. Using this method, they compare several FPs, including mStayGold, mBaoJin, and StayGold(E138D), and several mScarlet variants. The method proposed is interesting, but several questions remain before I can recommend publication.

Major comments:

The manuscript currently does not sufficiently acknowledge or contrast the presented methodology with existing approaches for assessing FP properties in live cells. For example, ratiometric analyses involving co-expression of FPs like mCherry to normalize expression levels are standard and should be discussed explicitly in the introduction. The authors should clarify why their approach is superior

© 2025. Published by The Company of Biologists under the terms of the Creative Commons Attribution License (https://creativecommons.org/licenses/by/4.0/).

or complementary to such methods. While the presented method is innovative, it would benefit from a discussion on scenarios where it may outperform or be less suitable than other methods.
Agreed. We revised the introduction to include discussion of ratiometric methods, which are really the only other method currently to objectively assess FP brightness directly in cells. In general, a drawback of ratiometric methods is that they assume that both FPs are present in the same amounts (i.e. same maturation time, same protein stability etc). Even with self-cleaving 2A peptide plasmids that is never directly demonstrated, and it is also likely that 2A peptides are not cleaved 100%, which could lead to photophysical interactions as mentioned by both reviewers. Generally, the fluorophore maturation times of FPs are well below 30 min, but the ratio between red and green FPs in these experiments frequently continues to change over days indicating that the dynamics of these ratiometric measurements are not fully understood. We propose nanocages as an alternative and complementary tool that does not rely on a ratio or expression levels because we are measuring the brightness of a specific number of molecules.

A critical question arises regarding the efficiency of nanocage assembly. How can the authors ensure that the nanocages are complete in cells? Presumably, the FP will fluoresce regardless of the number of subunits in the cage. Is the assembly uniform across the different FPs tested? The variability observed in StayGold(E138D) fluorescence intensity (Fig. 1F) suggests incomplete assembly or aggregation, which warrants further investigation. Please include quantitative data on the efficiency of the cage assembly and whether it varies with FP choice.
I3-01 nanocages are extremely stable. The original Hsia et al. paper demonstrates stability of a monodisperse population, i.e. fully assembled nanocages, in strongly denaturing conditions (6.7 M guanidinium hydrochloride). It is thus difficult to envision that nanocages once assembled would dissociate in cytoplasm. Consequently, the cytoplasmic background of our FP-tagged nanocage constructs is very low; to illustrate this we included a supplementary figure in which we boosted brightness (new suppl. Fig. S1A); it is impossible to see the edge of the cell indicating that there are almost no non-assembled nanocage constructs. [The apparent background in Fig. 1C is from rapidly diffusing nanocages in the absence of a hyperosmotic treatment]. In addition, a small amount of non-assembled FP-tagged nanocages in the cytoplasm does not impact our brightness measurements because the 2D Gaussian fit takes the local background into account and the integrated intensity is only the Gaussian peak above that local background (dark blue offset plane in Fig. 1F).
The Hsia et al. paper also demonstrated by gel filtration that there are no partial nanocages in an I3-01 protein preparation. Thus, while it is theoretically possible that there are some not fully assembled nanocages in our measured population as well as some nanocage doublets that are not resolved, these fractions would be expected to be very small. In contrast, we think that the variability of individual nanocage measurements stems mostly from variability in imaging (such as uneven illumination across the field; differences in focus; different amounts of scattered light contributing to variability in the local background etc) rather than an inherent variability of molecule numbers in nanocages. Consequently, the nanocage intensity standard deviation scaled linearly with the nanocage intensity mean for all red and green FPs tested (except for E138D; new suppl. Fig. S1B) indicating a similar relative variability, and hence a similar underlying nanocage population (of n = 300 particles each), that is thus independent of the FP-tag.
StayGold(E138D), however, is a clear outlier. The increased variability in E138D nanocage intensity indicates that E138D likely aggregates. Our goal was to find the best of the published mStayGold variants for our live imaging purpose and figuring out why E138D misbehaves or is poorly expressed in mammalian cells is outside the scope of this study.

It is also unclear how having sixty FPs in close proximity (in the cage) impacts the fluorescence properties of individual FPs. Could proximity-based quenching, Förster resonance energy transfer, or photophysical effects alter brightness or photostability?
The terminology nanocage seems to imply that FP molecules are inside the cage. That is not correct. The I3-01 peptides are all tagged at the N-terminus that points outside on recent published structures. It seems sterically impossible to pack 60 FPs into the inside of an I3-01 nanocage. It is correct that FPs are still relatively close to one another, but a quick calculation suggests that they are not within efficient FRET distance. I3-01 nanocages have a diameter of ~26 nm, roughly the size of a ribosome. GGS linkers that connect the FP to the I3-01 peptide are around 2-3 nm long and the FP molecule itself is around 4 nm long. Thus, in a conservative estimate, the centers of the 60 FP molecules are distributed on the surface of a sphere with a diameter of at least ~35 nm. Using a Fibonacci lattice to distribute these points evenly on the sphere (González, Á. Measurement of

© 2025. Published by The Company of Biologists under the terms of the Creative Commons Attribution License (https://creativecommons.org/licenses/by/4.0/).

Areas on a Sphere Using Fibonacci and Latitude-Longitude Lattices. Math Geosci 42, 49–64 (2010). https://doi.org/10.1007/s11004-009-9257-x), this results in an average spacing of FP molecules >7-8 nm from one another. While this is certainly close, it is further than 3-4 nm at which self-quenching or non-radiative energy transfer between FP molecules is thought to occur efficiently. Importantly, however, this is not just a theoretical inference, we also tested this in Fig. 2I by comparing the intensity of nanocages tagged with either 60 or 120 FP molecules. We show that the fluorescence intensity of mEmerald nanocages linearly doubles going from 60 to 120 molecules indicating that there is no self-quenching. In contrast, mStayGold molecules connected in tandem may be subject to a small degree of self-quenching (mStayGold nanocages with 120 mStayGold tags are slightly less bright than expected) and we acknowledge that in the text. However, this difference is small and does not affect our overall conclusions of which mStayGold variant is brightest.
In addition, if FPs were self-quenched around a nanocage, one would expect a delay in photobleaching (as fluorescent molecules disappear and previously quenched ones become fluorescent). This is not what we see in photobleaching experiments.

One major limitation of testing fluorescence properties in cells is that it does not always recapitulate the in vivo situation in tissues or whole organisms. How easily can the nanocage method be adapted for use in model organisms? Addressing the feasibility of this approach for in vivo studies would greatly broaden its impact.
We would argue that cytoplasm in cells in a dish is very similar to cytoplasm in cells in a tissue and thus, finding the optimal FP in tissue culture cells should be useful to make informed decisions for in vivo experiments as long as the comparison is done on the same microscope. In general, imaging deep into tissue is more difficult. The mStayGold nanocages however are exceptionally bright and it might be possible to image those in a 3D environment. However, this is outside the scope of the current study.

Minor Comments:

The title does not reflect the study as other FP than StayGold are tested.
We changed the title. This was a hold-over from when we initially had just compared GFPs.

Reviewer 2:
Giulia et al report on the comparison of monomeric StayGold variants in living cells. To this end they expressed fusion proteins that self-assemble into nanocages and they studied the brightness and photostability using spinning disk confocal microscopy. They report that mStayGold outperforms EGFP and is better than the E138D and mBaoJin monomeric versions of mStayGold.

1) Relevant papers are not cited that describe much more elaborate quantitative comparison of the 3 monomeric StayGold variants in cells which was published one year ago (Shimozono et al. (2024) BioRxiv, doi: 10.1101/2024.09.16.613379). Hence, their statement on page 3 "Although it is critical to select the brightest and most photostable fluorescent protein for live microscopy, no independent side-by-side comparison of these variants has been made" is misleading. Also not cited is the paper by Hirano et al (2024) Sci. Rep. 14:5541 which very elaborately descibes the behavior of StayGold under various illumination conditions in different microscopy implementations and show that the photostability is very dependent on the exact illumination conditions.
Note that both the above papers were published before the current study appeared online (February/March 2024 versus September 2024).
We apologize for not referencing the Shimozono preprint and we have corrected this oversight. We also cited the Hirano paper although it is not fully relevant here as it only compares the original dimeric StayGold to other GFPs and not the newer monomeric variants to one another. We fully agree that different microscope implementations affect the performance of specific fluorescent proteins and that is exactly why we wanted to provide a simple tool (and we will make the plasmids available) for us and others to do their own test on their specific equipment. We chose the spinning disk setup that we are using for live microscopy. Full spectroscopic analysis of bacterially expressed protein might be ‘more elaborate’ but possibly not that helpful for picking an FP for a specific microscopy application and was not what we set out here to do and as pointed out by the reviewer has been done by others.

© 2025. Published by The Company of Biologists under the terms of the Creative Commons Attribution License (https://creativecommons.org/licenses/by/4.0/).

Of note, the Shimozono preprint is from the same lab that initially described mStayGold. 'Independent' was meant to indicate a comparison of the three monomeric variants by someone other than the labs involved in the development of the monomeric variants. We think such an independent validation is crucial and worth reporting and in the spirit of reproducible science especially since we are using an alternative methodology. We rephrased this as to not offend anyone. Lastly, Shimozono et al. do not include any comparison to other GFPs.

2) The findings on mStaygold variants in essence confirm earlier published data: mStayGold is better than mBaoJin (equally bright but more photostable) and E138D (equally photostable but less bright), see Shimozono et al. (2024) BioRxiv, doi: 10.1101/2024.09.16.613379). So the overall novelty of the findings is questionable.
We corrected our oversight and are discussing the Shimozono preprint in the revised text. However, it is our understanding that replicating a result especially by using a completely different methodology is a good thing and should be published, and that JCS is an excellent journal to do so. Novelty of the overall conclusion is not relevant here, but novelty of providing an alternative method is.

3) mStayGold was reported to be sensitive towards tagging at the N-terminus (Ando et al, 2023). No sequence information is available about the fusion constructs used in the current study. For instance, the brightness of E138D can be greatly affected by small sequence additions at the N-terminus (Shimozono et al. (2024)). So likely the fusion to the N-terminus of the nanocage forming peptides will affect the brightness of some of the tested variants.
All nanocage constructs were tagged at the N-terminus of the nanocage peptide, not at the N-terminus of any of the FPs. The N-terminus (as well as the whole sequence) of all mStayGold variants tested is identical to sequences in the relevant three published papers; we now mention this explicitly in the text. So, the comment related to N-terminal sequences affecting brightness is not relevant here. We also included all plasmid maps as genebank files in the supplement. It should also be noted that there is little information about N-terminal sequences left behind after T2A cleavage in the Shimozono preprint.

4) The authors claim that the FP- nanocages allow quantitative comparison. Yet here it is assumed that a) the cages bringing many FPs in close proximity in an artificial environment do not change the properties of these FPs (for which no evidence is provided),
It is unclear why the reviewer insists that nanocages are an artificial environment. These nanocages are self-assembled protein structures that are tagged with the different FPs on the outside. Thus, FPs are exposed to the cytoplasm environment similar to any other tagged protein in a cell. For the purpose of evaluating FP performance in mammalian cytoplasm, this is more physiological than expression in bacteria or purified FPs in vitro.
I3-01 nanocages have a diameter of ~26 nm. GGS linkers that connect the FP to the I3-01 peptide are around 2-3 nm long and the FP molecule itself is around 4 nm long. Thus, in a conservative estimate, the centers of the 60 FP molecules are distributed on the surface of a sphere with a diameter of at least ~35 nm. Using a Fibonacci lattice to distribute these points evenly on a sphere (González, Á. Measurement of Areas on a Sphere Using Fibonacci and Latitude–Longitude Lattices. Math Geosci 42, 49–64 (2010). https://doi.org/10.1007/s11004-009-9257-x), this results in an average spacing of FP molecules >7 nm from one another. While this is certainly close, it is further than 3-4 nm at which self-quenching or non-radiative energy transfer between FP molecules is thought to occur efficiently.
Importantly, however, this is not just a theoretical inference, we also tested this in Fig. 2I by comparing the intensity of nanocages tagged with either 60 or 120 FP molecules. We show that the fluorescence intensity of mEmerald nanocages linearly doubles going from 60 to 120 molecules indicating that there is no self-quenching. In contrast, mStayGold molecules connected in tandem may be subject to a small degree of self-quenching (mStayGold nanocages with 120 mStayGold tags are slightly less bright than expected) and we acknowledge that in the text. However, this difference is small and does not affect our overall conclusions of which mStayGold variant is brightest.
In addition, if FPs were self-quenched around a nanocage, one would expect a delay in photobleaching (as fluorescent molecules disappear and previously quenched ones become fluorescent). This is not what we see in photobleaching experiments.

© 2025. Published by The Company of Biologists under the terms of the Creative Commons Attribution License (https://creativecommons.org/licenses/by/4.0/).

b) that all tested FPs have no residual tendency for oligomerization, influencing the size/number of FPs per nanocage (no evidence provided). In this respect I cite the authors on page 5, line 1: "StayGold(E138D) nanocage particle population was highly variable indicating either incomplete nanocage assembly or aggregation (Fig. 1F)". So here the quantitative brightness comparison is flawed as a result of this approach. Note for instance, that EGFP is a dimer since it contains an Alanine206.

We did consider aggregation of nanocages. We were especially concerned that the extraordinary brightness of mStayGold nanocages was due to aggregation. To this end, we compared the intracellular diffusion of mEmerald and mStayGold-tagged nanocages. The diffusion coefficient correlates with particle size and our measurement is sensitive enough to clearly distinguish nanocages with either 60 or 120 mStayGold molecules attached (Fig. 2D-F), and the difference in diffusion coefficient scaled with the expected size difference of these particles. In addition, the diffusion coefficients of mEmerald and mStayGold nanocages were indistinguishable, which demonstrates that they have the same size and thus that mStayGold nanocages do not aggregate.
Yes, we fully agree that there is a problem with E138D. We now include data (new suppl. Fig. S1B) showing that nanocage intensity means and standard deviations are linearly related indicating a constant relative variability independent of the FP-tag, with the exception of E138D that displayed a much higher variability. This is consistent with E138D aggregation. E138D was also very poorly expressed in mammalian cells. For these reasons we concluded that E138D is not a good choice for tagging proteins in mammalian cells. However, we did not further characterize why E138D performed poorly or aggregated as this was not the goal of the paper. The reviewer is correct that we might overestimate E138D brightness because of aggregation and we clarified this in the text.
I3-01 nanocages are extremely stable. The original Hsia et al. paper demonstrates stability of a monodisperse population, i.e. fully assembled nanocages, in strongly denaturing conditions (6.7 M guanidinium hydrochloride). It is thus difficult to envision that nanocages once assembled would dissociate in cytoplasm. This is supported by our own data showing very little cytoplasmic (i.e. soluble) background (new suppl. Fig. S1A).
If EGFP has a tendency to dimerize or not is not relevant to our conclusions. All comparisons of mStayGold variants are made relative to mEmerald. We included EGFP as well because it has been widely used by us and others to label all kinds of intracellular structures over many decades, and in our hands has never aggregated anything. This is fully consistent with our finding that the brightness of EGFP and mEmerald nanocages is indistinguishable.

5) The authors misleadingly state that "it is difficult to quantitatively compare FP characteristics in live cells" (page 5, line 42) and that these nanocages allow "compare the intrinsic brightness of FP variants on a molecule-by-molecule basis in physiologically relevant conditions" (page 1 line 56). For cell biologists these nanocages are irrelevant, since they are truly artificial non-physiological large assemblies within the cytoplasm.

Again, nanocages are protein assemblies that are tagged on the outside with the FPs under investigation. Their design is based on a bacterial aldolase trimer. They are about the size of a ribosome and freely diffuse in the cytoplasm, so the reviewer’s statement that nanocages are ‘artificial non-physiological large assemblies’ is simply not correct. By this logic, expression of anything in cells is artificial and hence irrelevant. This would invalidate the use of jellyfish fluorescent proteins or bacterial Cas9 in mammalian cells altogether. From our total cell intensity measurements (see modified Fig. 1H), we also estimate that the cells on the brighter end of the population we analyzed contain around 50,000 nanocages, which is ~2 orders of magnitude below the number of ribosomes in a typical mammalian cell, thus not a physiologically excessively high amount.
We do agree though that ‘intrinsic brightness’ is possibly misleading and that that terminology is better reserved for spectroscopic characterization of purified proteins. Here we use individual nanocage intensity measurements to compare FPs on a specific imaging system on a molecule-by-molecule basis and we revised the text accordingly.

Furthermore, in this study even no corrections are performed for expression level differences. They state that "a large variability is expected in transient transfections" (page 5, line 8) yet they continue to compare single transfected cells.

Yes, transient transfections are highly variable but the whole point of our approach is that we measure the intensity of individual nanocages, which always represent 60 molecules. The overall expression level is not important and while it changes the number of nanocage particles per cell, it does not alter the intensity of each individual nanocage. Maybe the reviewer misunderstood our

© 2025. Published by The Company of Biologists under the terms of the Creative Commons Attribution License (https://creativecommons.org/licenses/by/4.0/).

approach, and we tried to clarify this in the manuscript. The one experiment in which we measure total cell fluorescence is to show that the expression level distribution was similar in the population of EGFP-, mEmerald- and mStayGold-expressing cells we analyzed (but overall much lower in E138D-expressing cells; another reason why we think E138D does not work well in mammalian cells). We altered this panel to now show whole cell brightness relative to individual nanocage brightness (i.e. number of nanocages per cell; modified Fig. 1H), which better brings this point across (rather than mStayGold appearing to be much more highly expressed).

It is of note that in the study of Shimozono (2024) quantitative co-expression was done using 2A sequences that allow 1:1 production of diverse green FPs together with a red FP. Also elaborate brightness comparisons were done for all RFPs in which careful expression corrections and (in addition) spectral corrections accounting for different spectroscopic features were done both in living and in fixed cells (Gadella et al. 2023). Note that such data show that in cells mScarlet3 is >5 times brighter and not 2 times brighter than mCherry as is suggested in Supplemental Table I. These discrepancies likely reflect differences in transfection efficiency which are not corrected for.
We acknowledge that we failed to properly cite the Shimozono preprint and we now review intracellular ratiometric methods to assess FP brightness in the introduction. Both papers referenced by the reviewer use similar T2A / P2A self-cleaving peptide plasmids to co-express different wavelengths FPs. Of note, this method comes with its own assumptions that are not addressed by either of these papers. Frequently, 2A cleavage is inefficient and the two proteins remain connected. In this case, they are certainly close enough for FRET to occur, which would alter the measured fluorescence ratio. Similarly, ratiometric methods assume that both FPs mature similarly and have the same stability in cells. As far as we can determine neither of these papers addressed directly if the two FPs are in fact present at the same concentrations in cells. The observation that these ratios frequently drift over many hours or days even though FP maturation times are measured in minutes demonstrates that these ratio measurements are not as absolute as this reviewer likes us to believe and that having a complementary and alternative method that does not depend on ratios is important.
Yes, it was surprising and disappointing to us that mScarlet-3 was not that much brighter than mCherry. Again, transfection efficiency is irrelevant for our method as we measure and compare the absolute intensity of individual nanocages tagged with 60 FPs each. Instead, this may come down to differences in the spectral characteristics of the microscope systems used. While we used a long-pass emission filter to capture most of the light emitted by all the different red FPs, Gadella et al. employed a 593/46 band-pass filter that better matches the blue-shifted mScarlet emission spectrum. Together with a shorter excitation wavelength, in the raw data this overestimates the brightness of mScarlet3 relative to mCherry by at least 1.5-fold. Gadella et al. appear to correct for spectroscopic throughput of their imaging system, and we now do the same (in modified suppl. Table S1). Still, we only see a ~2-fold increase in mScarlet3 nanocage intensity compared with mCherry. We do not understand where this discrepancy comes from but do not have any reason to believe that our measurements are incorrect.
Ultimately, a researcher will mostly care about how a specific FP performs on their imaging setup and these unexpected differences once again highlight the importance of testing FPs on the microscope used for a specific experiment. We propose FP-tagged nanocages as a simple and alternative tool to test this that also avoids potential complications of ratiometric methods and allows to compare absolute FP intensity.

6) No corrections are done for spectroscopic throughput. Despite differences in excitation/emission spectra in EGFP, mEmerald and the three mStayGolds. So, the numbers obtained are biased by the employed excitation lines and emission filters in the microscopy setup.
In the earlier version of this manuscript, we did correct for differences in excitation peak, which is especially relevant for the red-shifted StayGold excitation spectra. We did not initially correct for differences in emission spectra because our emission channels captured very similar fractions of green and red FP emission spectra, respectively. It does make a small difference (mostly for the mStayGolds), and we included this now in the last column of modified suppl. Table S1 and modified Fig. 4.

7) The illumination power used for all experiments (15 W/cm2), page 19, line 42, is abnormally high and not in any way relevant for regular cell biological fluorescence microscopy experiments.
No, this is not abnormally high. While we generally strive to minimize irradiance in live imaging experiments to minimize photobleaching and toxicity, here we chose to be a little on the high side

© 2025. Published by The Company of Biologists under the terms of the Creative Commons Attribution License (https://creativecommons.org/licenses/by/4.0/).

to image nanocages with SoRa super-resolution spinning disk confocal microscopy with an effective 27 nm pixel size. High SNR data at this high effective magnification require a slightly higher power density than we would normally use for live cell imaging (see https://pubmed.ncbi.nlm.nih.gov/24974023/ for some basic calculations). This also helps with observing photobleaching at a shorter timescale. Of note, the Shimozono preprint uses 8.66 W cm^{-2} irradiance for photostability measurements, which is not that different from our experiments (and they do not report the irradiance in their brightness measurements). This irradiance is well below FP ground-state depletion. Thus, both brightness and photostability are nearly linearly related to irradiance. In addition, laser scanning microscopes exceed this irradiance by orders of magnitude. Gadella et al. report a laser power of 3 μW at the focal plane in their live laser scanning confocal experiments. With an estimated Airy disk radius of 300 nm, this yields an irradiance of ~1000 W cm^{-2}. The original Ando et al. mStayGold paper uses 10 – 1000 W cm^{-2} irradiances to measure photobleaching. Thus, 15 W cm^{-2} is well within the range that one uses for live imaging experiments and these types of measurements.

8) The maturation of these FPs in cells is a characteristic of major importance and relevance for cell biologists but completely neglected in the current study. Both the speed and final extent of chromophore maturation are critical parameters for comparing FPs. While these parameters are tested extensively for these FPs in other studies (see Ando et al., 2023, Shimozono et al. (2024) Gadella et al (2023), no information is found on this in the current comparison.
I would disagree that maturation times are that relevant for live microscopy. What we really care about is brightness and photostability on a given imaging setup, and our manuscript provides a new tool to measure and compare these parameters directly on the specific imaging system used. Maturation of the original dimeric StayGold was reported as 14 min. The maturation times reported on FPbase for EGFP (25 min) and mEmerald (11 min) are in a similar range. We cannot actually find measured maturation times for mStayGold in the Shimozono preprint or the original Ando paper. The reported value for mBaoJin is 8 min.
Thus, compared to the time scale of imaging (24-48 hours after transfection), these maturation times are short and would at most account for 1-2% of FP molecules not being fluorescent at equilibrium. So, yes, it is possible that we are only measuring the fluorescence of 59 molecules per nanocage but this is a very small error and does not invalidate any of our conclusions.
Given these relative short maturation times the ratiometric brightness curves reported in Shimozono Fig. 3 and in the original mStayGold Ando et al. paper are difficult to interpret. Based on fluorophore maturation alone, it does not make sense that the ratio normalized to mCherry (with a maturation time of 15 min) would continue to increase up to 48 hours after transfection. This indicates that something else is happening here that we do not fully understand (e.g. a change in relative protein amounts over time) and indicates a flaw of this ratiometric method and the need for an alternative and complementary tool.

Second decision letter

MS ID#: jcs.263858R1

MS Title: Quantitative Comparison of Monomeric StayGold Variants Using Protein Nanocages in Living Cells

Authors: Giulia Viola; Yasmeen Ibrahim; Kyle A Jacobs; Joel Lemiere; Matthew L Kutys; Torsten Wittmann
Article Type: Tools and Resources

Dear Dr Wittmann,

Thank you for your recent letter. I understand how disappointed you must feel.

Given the opinions stated by the reviewers, I had no choice but to reject the paper.

© 2025. Published by The Company of Biologists under the terms of the Creative Commons Attribution License (https://creativecommons.org/licenses/by/4.0/).

However, we are always willing to give authors the chance to defend their manuscripts. In the light of the comments you make in your letter, I have decided to proceed as follows. I would be happy for you to submit a revised version of your manuscript that deals as far as possible with the points raised by the reviewers, together with a detailed rebuttal of any other matter that cannot be settled in the manuscript itself. I will then send the revised version and the rebuttal back to the reviewers. If they are convinced by your arguments, then we would be happy to accept the manuscript for publication.

Third decision letter

MS ID#: jcs.263858R2

MS Title: Quantitative Comparison of Fluorescent Proteins using Protein Nanocages in Live Cells

Authors: Giulia Viola; Yasmeen Ibrahim; Kyle A Jacobs; Joel Lemiere; Matthew L Kutys; Torsten Wittmann
Article Type: Tools and Resources

Dear Torsten,

After consulting with a member of the advisory board and in light of your full response to the reviewers questions, I am happy to tell you that your manuscript has been accepted for publication in Journal of Cell Science, pending standard publication integrity checks. It was accepted on the 25th of April. Where referee reports on this version are available, they are appended below.

© 2025. Published by The Company of Biologists under the terms of the Creative Commons Attribution License (https://creativecommons.org/licenses/by/4.0/).
